# Supplementary material for: Loss of AXIN1 drives acquired resistance to WNT pathway blockade in colorectal cancer cells carrying RSPO3 fusions
Source: EMBO Mol Med. 2017 Jan 18;9(3):293–303. doi: 10.15252/emmm.201606773 (PMC5331210; doi:10.15252/emmm.201606773)
Supplement: Supplementary file 1 — Appendix [file EMMM-9-293-s001.pdf]

# Loss of Axin1 drives acquired resistance to WNT pathway blockade in colorectal cancers cells carrying RSPO3 fusions.

Gabriele Picco[1,2], Consalvo Petti[1,2], Alessia Centonze[2], Erica Torchiaro[1], Giovanni Crisafulli[1], Luca Novara[1], Andrea Acquaviva[3], Alberto Bardelli[1,2] and Enzo Medico*[1,2]

# APPENDIX

## Table of contents

**Appendix Figure S1.** Stromal scores vs. RSPO3 expression in the TCGA dataset.

**Appendix Figure S2.** RSPO3 expression vs. stromal scores in a 2140-sample CRC microarray dataset.

**Appendix Figure S3.** Absence of RSPO3 rearrangements in 12 additional TCGA CRC samples.

**Appendix Figure S4.** Validation by Sanger sequencing of RSPO3 fusion transcripts in CRC cell lines.

**Appendix Figure S5.** Axin1 mutations detected in $VACO6_R$.

**Appendix Figure S6.** Single clone analysis of VACO6R cells.

**Appendix Figure S7.** $VACO6_R$ cells are specifically resistant to WNT pathway inhibitors.

**Appendix Table S1A.** RSPO2/3 fusion analysis in TCGA CRC samples.

**Appendix Table S1B.** Settings of Defuse, Chimerascan and Mapsplice tools.

**Appendix Table S2A.** Point mutations detected by exome sequencing of $VACO6_R$ cells.

**Appendix Table S2B.** Insertions / Deletions detected by exome sequencing of $VACO6_R$.

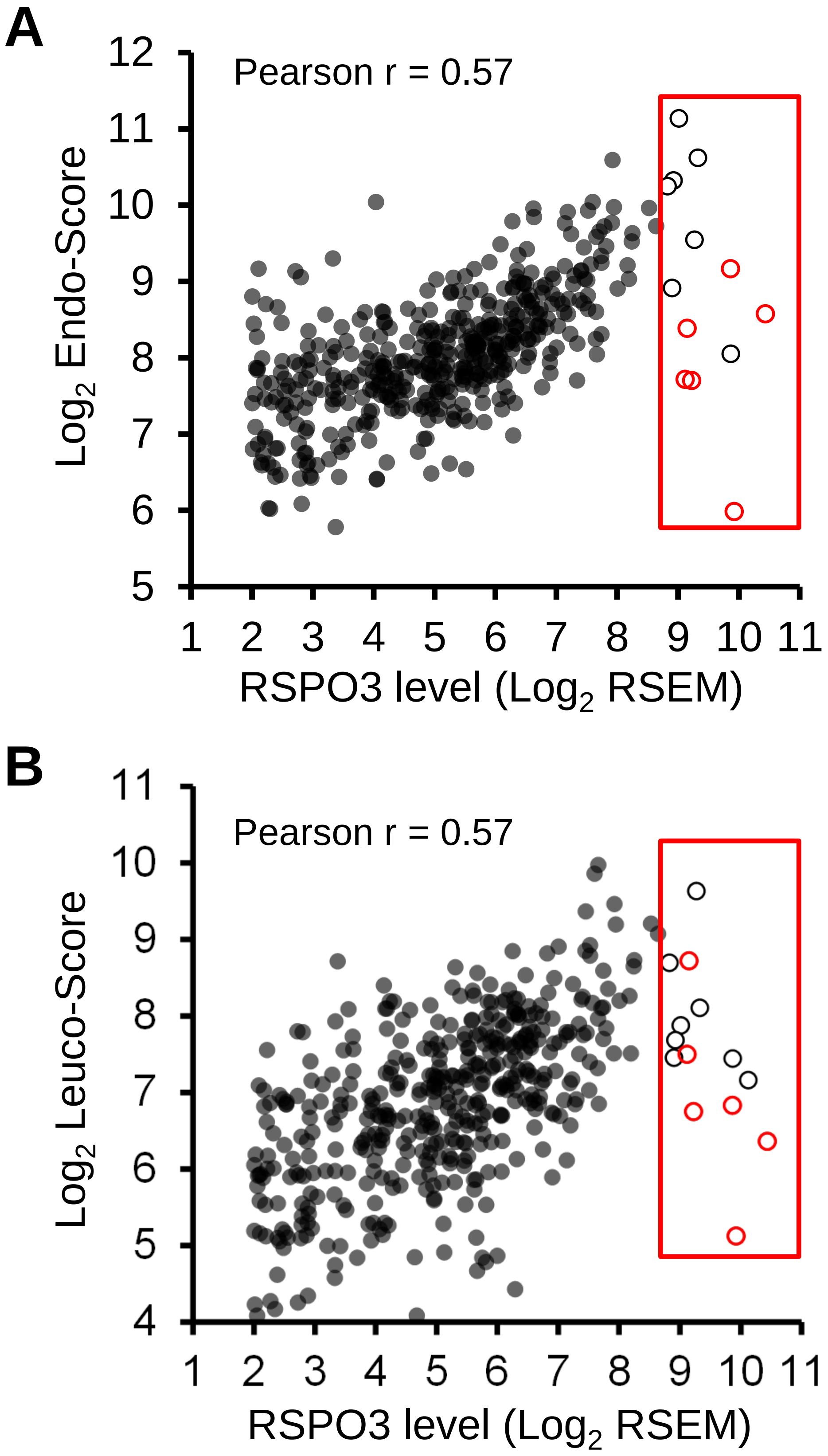


**Picco et al., Appendix Figure S1. Stromal scores vs. RSPO3 expression in the TCGA dataset.** Scatter plots displaying the correlation between RSPO3 expression levels (x-axis) and Endo-score (A) or Leuco-score (B). The red boxes highlight samples with high RSPO3 expression and variable Endo- or Leuco-score. Empty dots indicate samples selected for fusion analysis. Empty dots with red border indicate samples with high RSPO3 levels and medium-low CAF-score, also highlighted in Figure 1A.

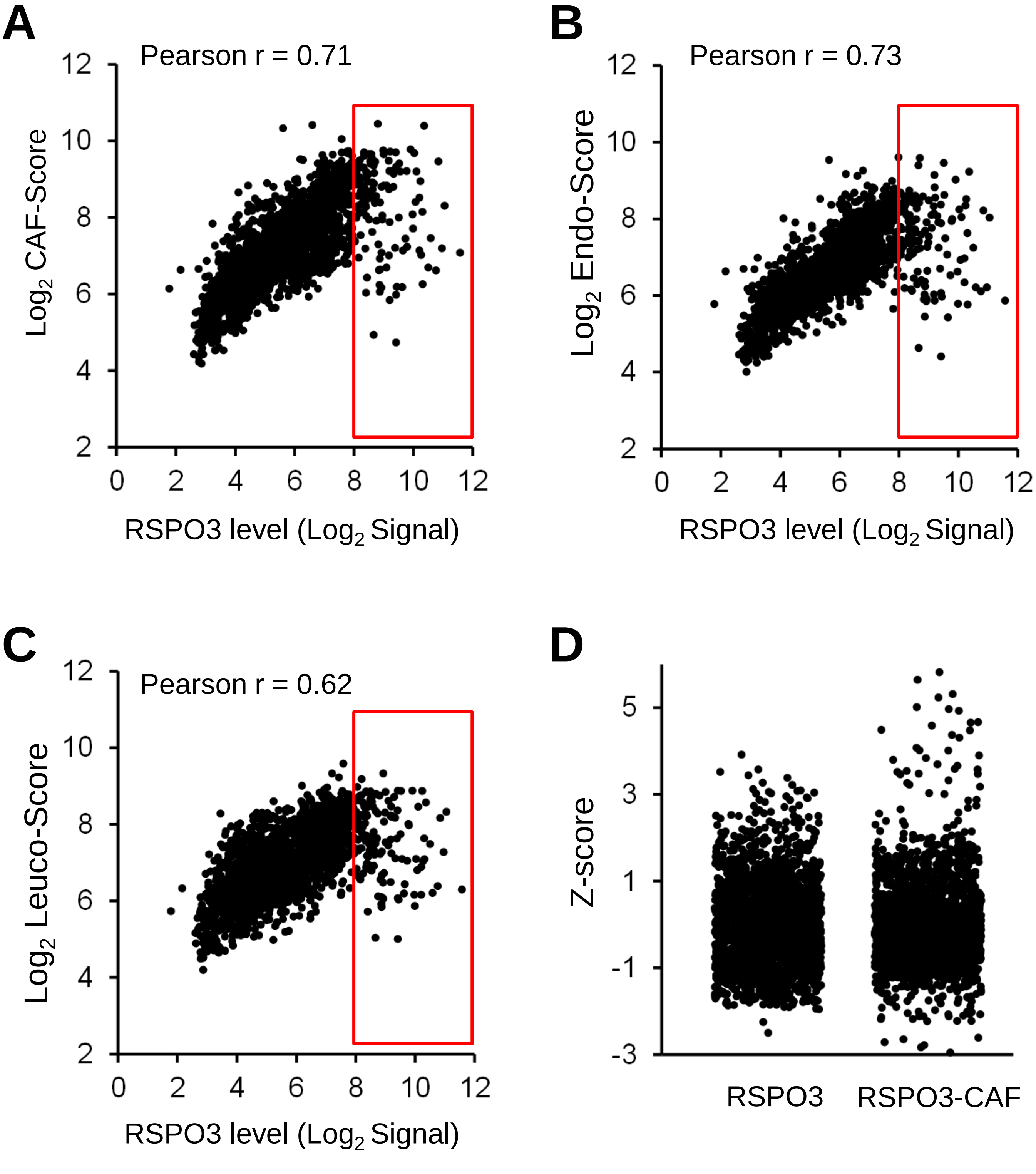


**Picco et al., Appendix Figure S2. RSPO3 expression vs. stromal scores in a 2140-sample CRC microarray dataset.** A composite CRC gene expression dataset was built from the following public datasets: GSE13294, GSE14333, GSE17536, GSE20916, GSE2109, GSE35896, GSE37892, GSE39582, KFSYSCC. Scatter plots display the correlation between RSPO3 expression levels on the x-axis and CAF-score (A), Endo-score (B) and Leuco-score (C), on the y-axis. The red boxes highlight samples with high RSPO3 expression and variable stromal scores. (D) Dot plots displaying the distribution of Z-Score values for RSPO3 alone and RSPO3 minus CAF-score.

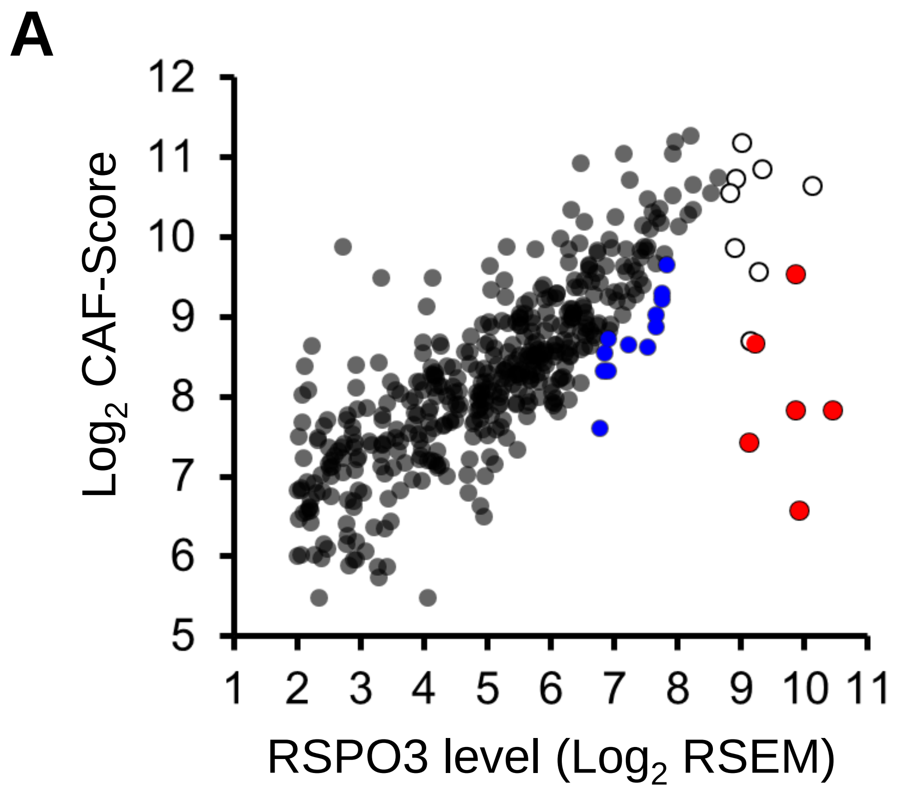


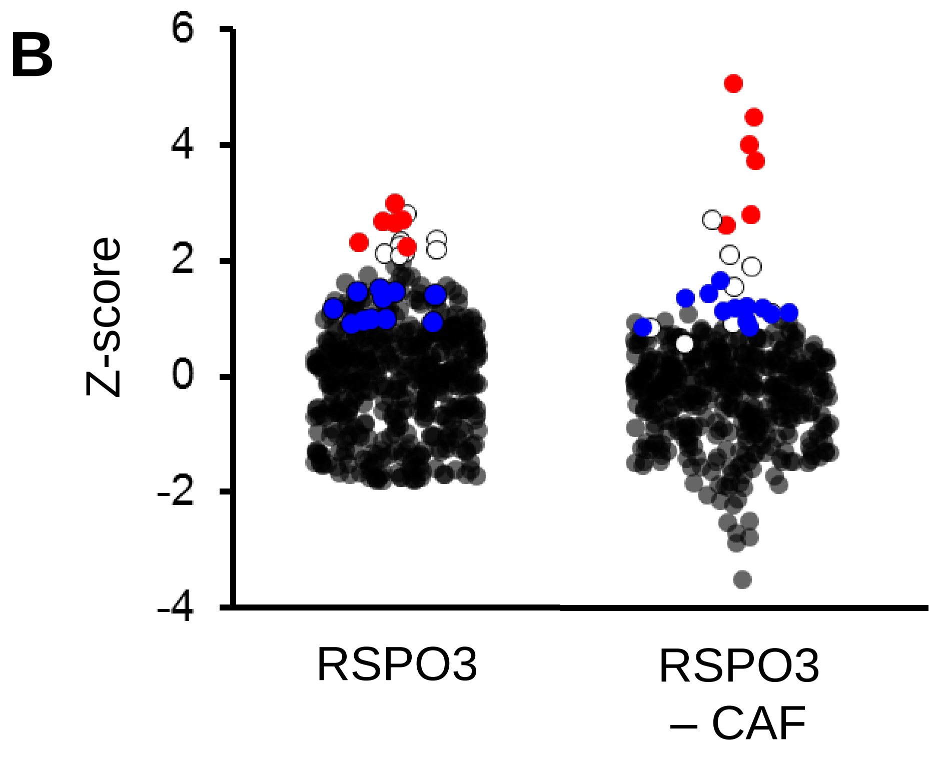


**Picco et al., Appendix Figure S3. Absence of RSPO3 rearrangements in 12 additional TCGA CRC samples**. (A) Scatter plot displaying the correlation between RSPO3 expression (x-axis) and CAF-score (y-axis). (B) Dot plots displaying the distribution of Z-Score values for RSPO3 alone (left panel) and RSPO3 minus CAF-score (right panel) in the 450-sample TCGA dataset. Blue dots indicate 12 samples with intermediate RSPO3 expression and low CAF-score, selected to complete the search for RSPO3 fusions. Empty dots indicate previously analyzed samples that do not carry RSPO3 fusions. Red dots indicate RSPO3 fused samples.

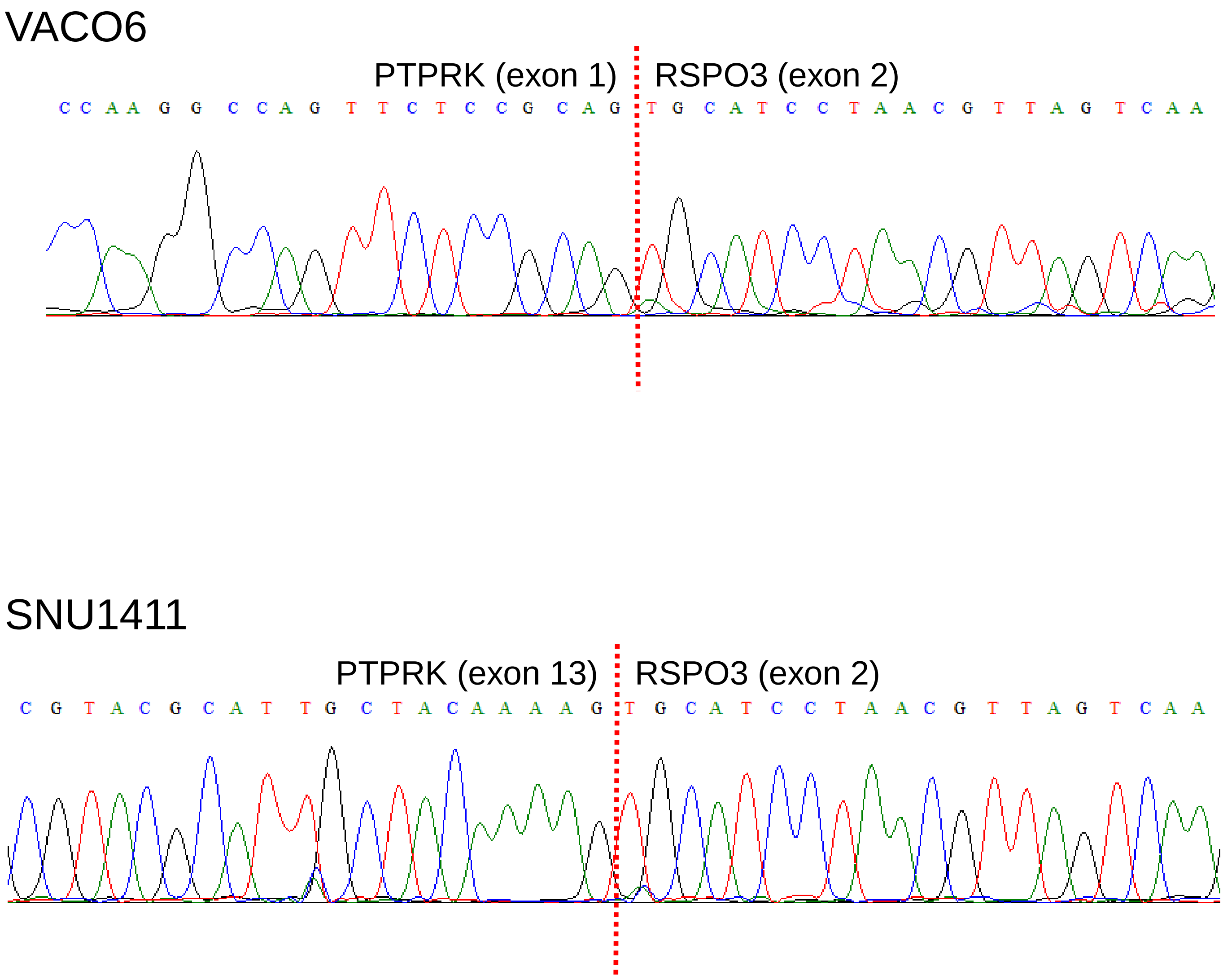


**Picco et al., Appendix Figure S4: validation by Sanger sequencing of RSPO3 fusion transcripts in CRC cell lines.** Representative Sanger sequencing chromatograms of RT–PCR amplicons confirming the PTPRK(e1)–RSPO3(e2) and the novel PTPRK(e13)–RSPO3(e2) fusion junctions in VACO6 and SNU1411 cells, respectively.

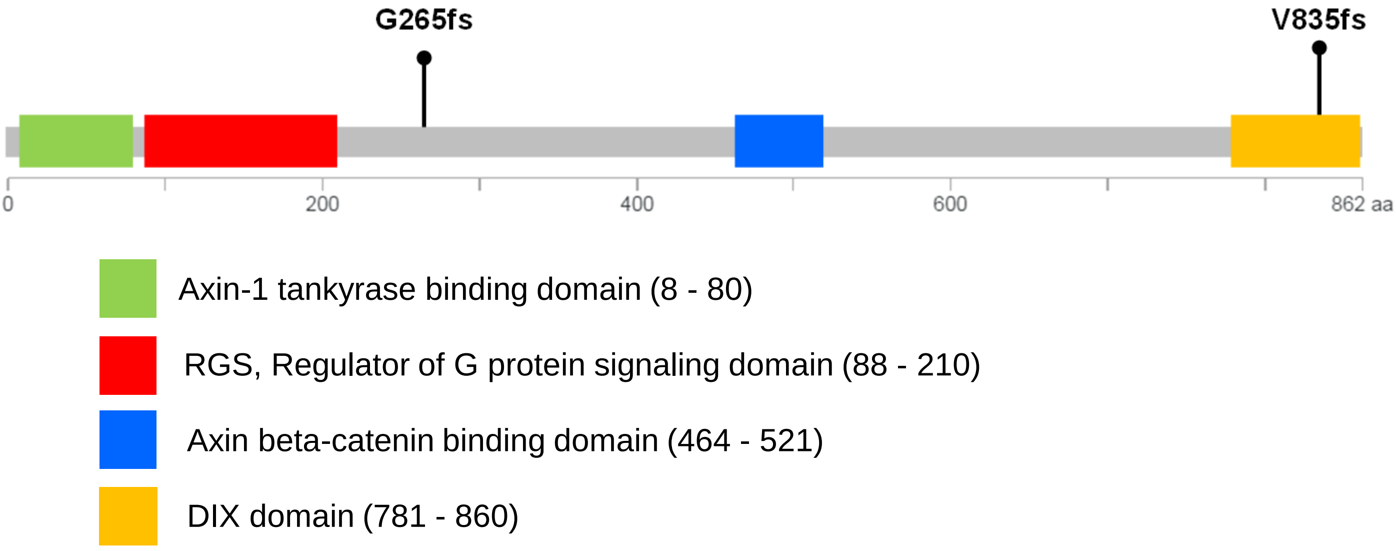


**Picco et al., Appendix Figure S5. Axin1 mutations detected in VACO6$_R$.** Representation of the two frameshift-inducing single nucleotide deletions in the AXIN1 gene in VACO6$_R$ cells, as reported for human tumor samples by cBioPortal (www.cbioportal.org; Cerami et al., Cancer Discov. 2012). Color-coded rectangles highlight AXIN1 protein domains.

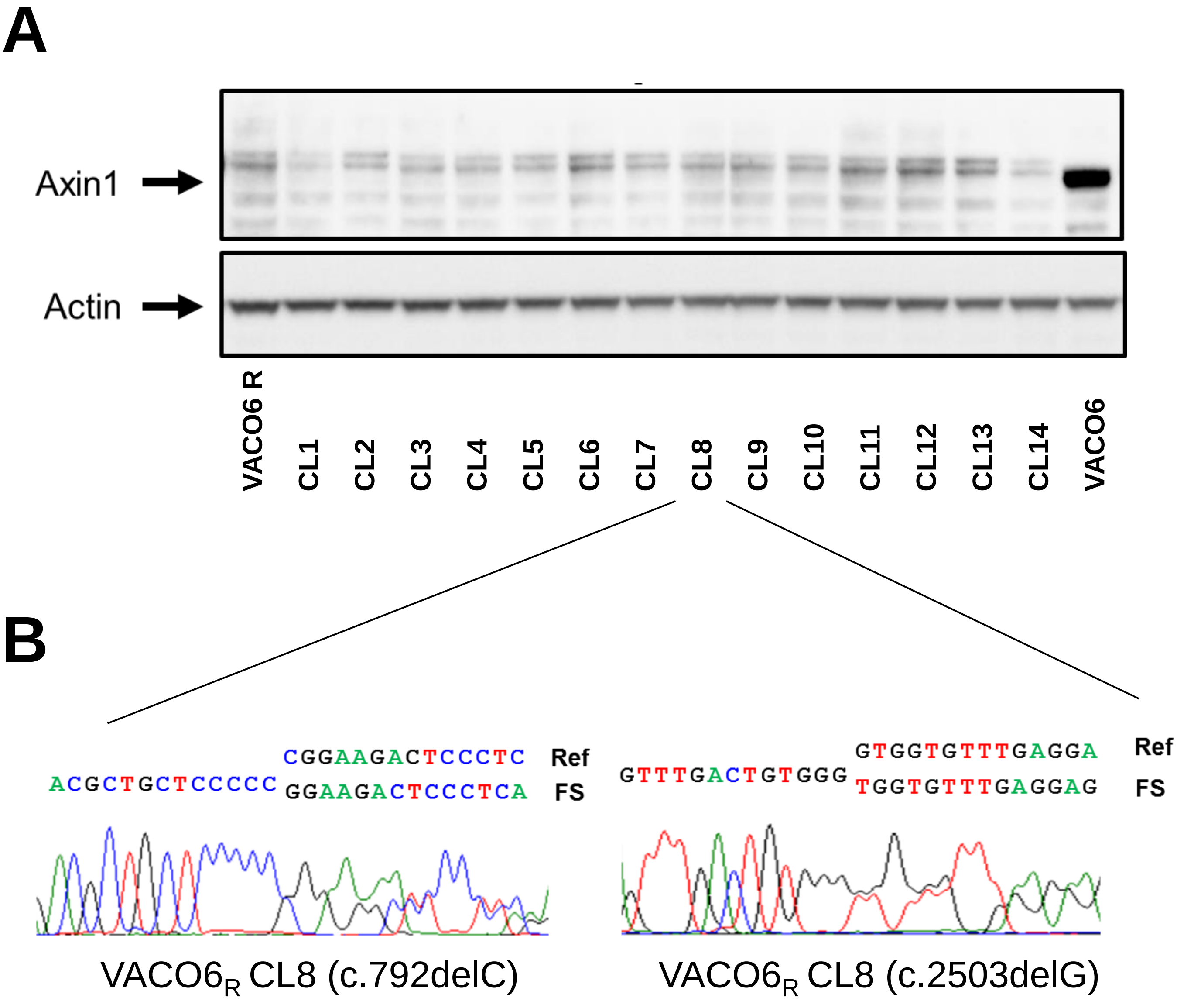


**Picco et al., Appendix Figure S6. Single clone analysis of VACO6$_R$ cells.**
(A) Western blot showing loss of the Axin1 protein in all 14 single cell clones derived from VACO6$_R$ population. (B) Representative chromatograms of Sanger-sequenced PCR products showing the two c.792delC and c.2503delG AXIN1 frameshift deletions in a representative clone (CL8) derived from VACO6$_R$ cells.

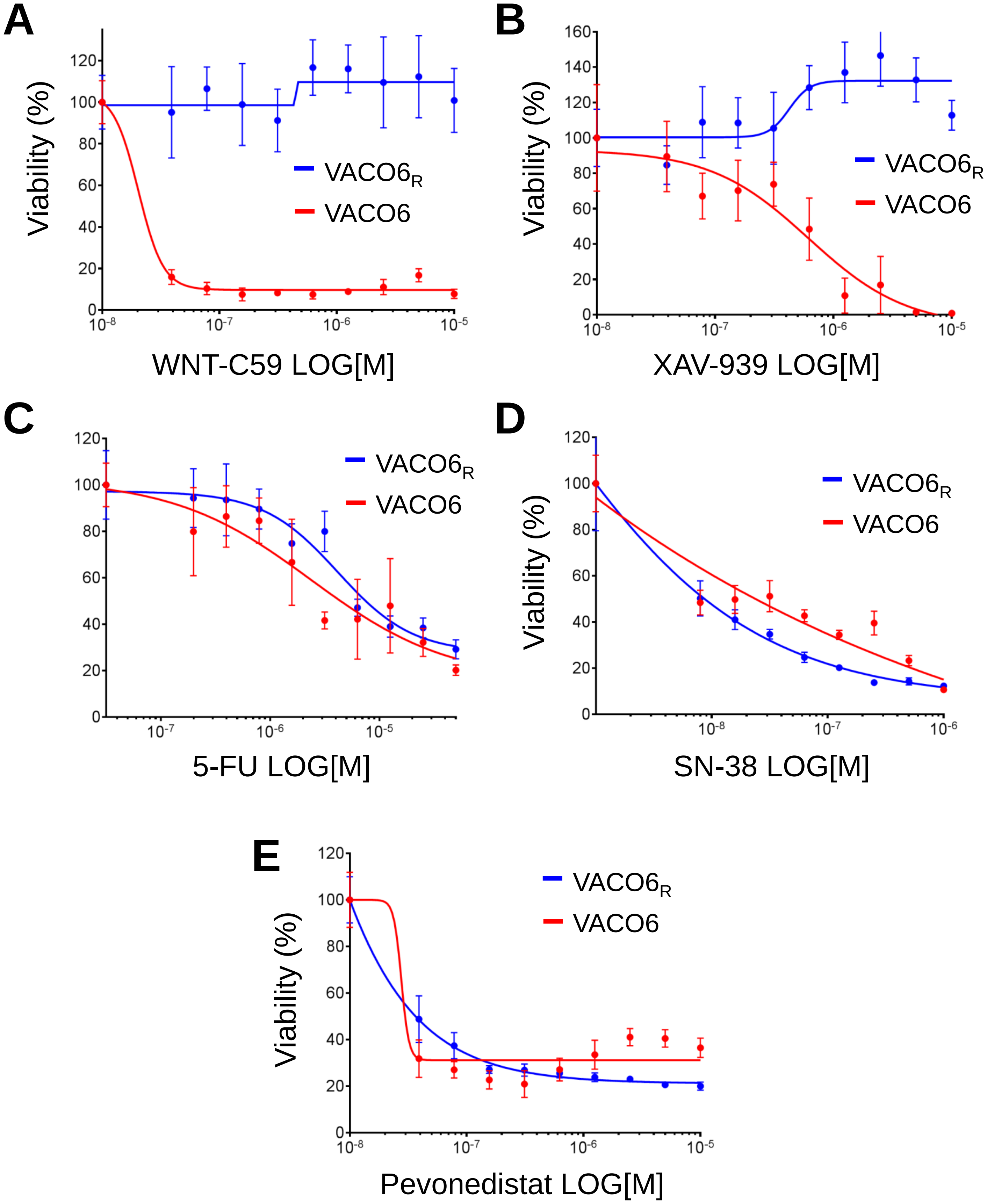


**Picco et al., Appendix Figure S7. VACO6$_R$ cells are specifically resistant to WNT pathway inhibitors.** ATP-based assay measuring viability of VACO6 and VACO6$_R$ cells after 7 days of treatment with WNT-C59 (A), XAV-939 (B), 5-FU (C), SN-38 (D) and Pevonedistat (E). Data are expressed as average ± s.d. of six technical replicates

**Picco et al. Appendix Table S1A. RSPO2/3 fusion analysis in TCGA CRC samples.** The table reports (i) the ID of CRC TGCA samples selected for the analysis (ii) the APC, KRAS and BRAF mutational status (iii) the RSPO3 mRNA levels; (iv) the CAF-signature values; (v) the level of stromal contamination, (vi) the Z-score after CAF-score correction (vii) the type of fusion trascripts identified, (viii) the tools that indentified the fusion, (ix) the number of reads supporting the fusion and (x) the type of RNA-seq reads available for the analysis.

| TCGA sample ID | APC status | KRAS status | BRAF status | RSPO3 expression ($Log_2$ RSEM) | RSPO3 expression ($Log_2$ RSEM) Z-score | CAF-score (Log2) | Delta (RSPO3 - CAF-score) | Delta (RSPO3 - CAF-score) Z-score | RSPO3 fusion | Tools that identified the fusion | Number of supporting reads | RNA-seq reads type |
|---|---|---|---|---|---|---|---|---|---|---|---|---|
| TCGA-CA-5255 | | | | 9.93 | 2.71 | 6.58 | **3.35** | 5.58 | PTPRK(ex1)-RSPO3(ex2) | defuse, mapsplice | 7 | paired end |
| TCGA-AF-6136 | | | | 10.44 | 3.00 | 7.82 | **2.61** | 4.95 | PTPRK(ex1)-RSPO3(ex2) | chimerascan, mapsplice | 5 | paired end |
| TCGA-D5-6539 | | | | 9.86 | 2.67 | 7.83 | **2.03** | 4.45 | PTPRK(ex1)-RSPO3(ex2) | chimerascan | 10 | paired end |
| TCGA-AA-3518 | WT | WT | WT | 9.12 | 2.25 | 7.43 | **1.69** | 4.15 | PTPRK(ex1)-RSPO3(ex2) | mapsplice | 5 | single end |
| TCGA-AA-3842 | WT | MUT:G13D | WT | 9.23 | 2.31 | 8.67 | **0.56** | 3.18 | PTPRK(ex1)-RSPO3(ex2) | mapsplice | 3 | single end |
| TCGA-A6-2672 | WT | WT | MUT:V600E | 9.15 | 2.27 | 8.70 | **0.45** | 3.08 | not detected | | | paired end |
| TCGA-DT-5265 | | | | 9.87 | 2.67 | 9.54 | **0.33** | 2.98 | PTPRK(ex1)-RSPO3(ex2) | defuse | 3 | paired end |
| TCGA-AZ-4323 | | | | 9.27 | 2.34 | 9.56 | **-0.29** | 2.45 | not detected | | | paired end |
| TCGA-AA-3695 | MUT:R213* | MUT:G12D | WT | 10.12 | 2.82 | 10.65 | **-0.53** | 2.24 | not detected | | | single end |
| TCGA-F5-6812 | | | | 8.90 | 2.13 | 9.86 | **-0.96** | 1.87 | not detected | | | paired end |
| TCGA-CM-6167 | | | | 9.33 | 2.37 | 10.85 | **-1.52** | 1.38 | not detected | | | paired end |
| TCGA-A6-6651 | | | | 8.83 | 2.09 | 10.55 | **-1.72** | 1.21 | not detected | | | paired end |
| TCGA-G4-6302 | | | | 8.93 | 2.14 | 10.74 | **-1.81** | 1.14 | not detected | | | paired end |
| TCGA-EI-7004 | | | | 9.02 | 2.19 | 11.19 | **-2.17** | 0.83 | not detected | | | paired end |

| **Picco et al. Appendix Table S1B.** Settings for fusion transcript search in RNAseq data with Defuse, Chimerascan and Mapsplice tools. | |
|---|---|
| ***tool*** | ***parameters*** |
| **defuse (version 0.6.1)** | # Bowtie parameters<br>bowtie_threads = 1<br>bowtie_quals = --phred33-quals<br>max_insert_size = 500<br><br># Blat sequences per job<br>num_blat_sequences = 10000<br><br># Minimum gene fusion range<br>dna_concordant_length = 2000<br><br># Trim length for discordant reads (split reads are not trimmed)<br>discord_read_trim = 50<br><br># Filtering parameters<br>clustering_precision = 0.95<br>span_count_threshold = 5<br>percent_identity_threshold = 0.90<br>split_min_anchor = 4<br>splice_bias = 10<br>probability_threshold = 0.50 |
| **chimerascan (version 0.4.5)** | -p 32 --library-type fr-firststrand --multihits 50 --initial-mismatches 1 --discord-mismatches 2 --segment-length 40 --homology-mismatches 1 --anchor-min 6 --anchor-length 12 --filter-unique-frags 4.0 |
| **mapsplice (version 2.1.8)** | --fusion-non-canonical --threads 32 |

**Appendix Table S2A. Point mutations detected by exome sequencing of VACO6$_R$ cells compared to VACO6 cells.**

| GENE ID | Cohordinates | Nucleotide change | Effect on translation | AA change | Position depth VACO6 | Position depth VACO6$_R$ | % mutant reads | Occurrences in Cosmic |
|---|---|---|---|---|---|---|---|---|
| CDK18 | chr1:205498487 | c.G1198A | nonsyn. | p.A400T | 164 | 115 | 43 | 2 |
| PNKP | chr19:50365045 | c.G1282T | nonsyn. | p.A428S | 333 | 296 | 38 | 2 |
| AP1M1 | chr19:16339599 | c.C943T | nonsyn. | p.R315W | 128 | 158 | 37 | 2 |
| OCM2 | chr7:97619395 | c.A22T | nonsyn. | p.S8C | 101 | 115 | 43 | 1 |
| MAP4K3 | chr2:39553363 | c.G586T | nonsyn. | p.D196Y | 128 | 135 | 7 | 1 |
| SDHA | chr5:256455 | c.C1915G | nonsyn. | p.L639V | 320 | 281 | 4 | 1 |
| ZCCHC12 | chrX:117960084 | c.C877T | nonsyn. | p.P293S | 113 | 103 | 79 | 0 |
| NXF3 | chrX:102334704 | c.G1147A | nonsyn. | p.E383K | 51 | 53 | 66 | 0 |
| COL9A3 | chr20:61452558 | c.C335A | nonsyn. | p.P112H | 172 | 151 | 55 | 0 |
| AIF1L | chr9:133995659 | c.C481G | nonsyn. | p.P161A | 100 | 95 | 52 | 0 |
| ZNF134 | chr19:58132760 | c.G1273T | nonsyn. | p.G425C | 115 | 98 | 51 | 0 |
| ARHGAP21 | chr10:24874647 | c.T4571C | nonsyn. | p.L1524P | 137 | 108 | 48 | 0 |
| DLGAP3 | chr1:35370359 | c.A626T | nonsyn. | p.D209V | 111 | 129 | 47 | 0 |
| EIF4G1 | chr3:184049311 | c.C4333T | nonsyn. | p.L1445F | 298 | 290 | 46 | 0 |
| LCA5L | chr21:40795085 | c.G654T | nonsyn. | p.Q218H | 134 | 152 | 45 | 0 |
| NOL8 | chr9:95078363 | c.T544C | nonsyn. | p.F182L | 75 | 94 | 45 | 0 |
| SORCS3 | chr10:106401117 | c.G32A | nonsyn. | p.G11D | 87 | 77 | 44 | 0 |
| FLRT3 | chr20:14307220 | c.C933A | stopgain | p.C311* | 165 | 167 | 43 | 0 |
| SRRD | chr22:26879962 | c.G106C | nonsyn. | p.G36R | 19 | 25 | 40 | 0 |
| AP1M1 | chr19:16339598 | c.G942T | nonsyn. | p.K314N | 122 | 146 | 40 | 0 |
| ANKRD6 | chr6:90340265 | c.C1726A | nonsyn. | p.Q576K | 178 | 186 | 39 | 0 |
| SCFD2 | chr4:54231369 | c.C740T | nonsyn. | p.A247V | 116 | 140 | 39 | 0 |
| SEMA6B | chr19:4558147 | c.C136T | nonsyn. | p.P46S | 37 | 39 | 38 | 0 |
| LRRC10B | chr11:61276987 | c.G517T | stopgain | p.E173* | 151 | 127 | 38 | 0 |
| FUK | chr16:70506992 | c.C1513A | nonsyn. | p.L505M | 130 | 120 | 38 | 0 |
| ADH6 | chr4:100131372 | c.G434A | nonsyn. | p.S145N | 215 | 259 | 37 | 0 |
| CYP46A1 | chr14:100191750 | c.G1199A | nonsyn. | p.R400Q | 104 | 77 | 36 | 0 |
| NUTM2F | chr9:97081325 | c.G1693T | nonsyn. | p.V565L | 118 | 111 | 36 | 0 |
| SEC16B | chr1:177927999 | c.G1110T | nonsyn. | p.L370F | 75 | 93 | 34 | 0 |
| AKAP9 | chr7:91708592 | c.A7145T | nonsyn. | p.D2382V | 240 | 230 | 34 | 0 |
| TTN | chr2:179429009 | c.G81850A | nonsyn. | p.V27284I | 248 | 256 | 34 | 0 |
| AKAP9 | chr7:91708591 | c.G7144A | nonsyn. | p.D2382N | 257 | 247 | 33 | 0 |
| LPPR3 | chr19:813427 | c.G1384A | nonsyn. | p.A462T | 158 | 101 | 32 | 0 |
| LYPD3 | chr19:43965642 | c.C902A | nonsyn. | p.A301D | 222 | 210 | 31 | 0 |
| KIAA0430 | chr16:15729770 | c.C574T | nonsyn. | p.P192S | 251 | 211 | 31 | 0 |
| POU5F1 | chr6:31138186 | c.G212T | nonsyn. | p.G71V | 205 | 200 | 31 | 0 |
| KCNK15 | chr20:43379012 | c.G526A | nonsyn. | p.V176I | 265 | 192 | 30 | 0 |
| RASGRF1 | chr15:79291149 | c.G2813T | nonsyn. | p.R938M | 207 | 207 | 29 | 0 |
| RBM15B | chr3:51430792 | c.C1962A | nonsyn. | p.S654R | 131 | 124 | 25 | 0 |
| AKAP3 | chr12:4737115 | c.T953C | nonsyn. | p.L318P | 272 | 269 | 21 | 0 |
| RBM15B | chr3:51430809 | c.C1979T | nonsyn. | p.A660V | 129 | 111 | 21 | 0 |
| LMO1 | chr11:8251938 | c.G139A | nonsyn. | p.D47N | 205 | 254 | 20 | 0 |
| MRPL42 | chr12:93870791 | c.T132G | nonsyn. | p.N44K | 117 | 121 | 18 | 0 |
| SLC2A3 | chr12:8075523 | c.T1166C | nonsyn. | p.V389A | 202 | 219 | 15 | 0 |
| ABCA4 | chr1:94496009 | c.C4327T | nonsyn. | p.R1443C | 333 | 341 | 14 | 0 |
| OLFML2B | chr1:161993133 | c.G88A | nonsyn. | p.E30K | 70 | 81 | 12 | 0 |
| NUTM2G | chr9:99700853 | c.G1648T | nonsyn. | p.V550L | 368 | 342 | 9 | 0 |
| LRRC15 | chr3:194081462 | c.G329A | nonsyn. | p.R110H | 178 | 193 | 9 | 0 |
| ZFP64 | chr20:50701323 | c.G1711C | nonsyn. | p.V571L | 225 | 204 | 9 | 0 |
| TTN | chr2:179658207 | c.A1460T | nonsyn. | p.K487M | 124 | 149 | 9 | 0 |
| FGG | chr4:155529781 | c.T688A | nonsyn. | p.F230I | 141 | 145 | 8 | 0 |
| CACNA1C | chr12:2711030 | c.A2924G | nonsyn. | p.Y975C | 331 | 321 | 8 | 0 |
| KRT81 | chr12:52681013 | c.G1120A | nonsyn. | p.A374T | 584 | 512 | 8 | 0 |
| SMG7 | chr1:183495830 | c.G412A | nonsyn. | p.A138T | 131 | 143 | 8 | 0 |
| CACNA1C | chr12:2774080 | c.C4466A | nonsyn. | p.T1489K | 339 | 323 | 7 | 0 |
| GCNT2 | chr6:10529656 | c.T512C | nonsyn. | p.L171P | 189 | 177 | 7 | 0 |
| TRIM26 | chr6:30154018 | c.T1255G | nonsyn. | p.L419V | 117 | 155 | 7 | 0 |
| WDR35 | chr2:20131104 | c.G2923A | nonsyn. | p.A975T | 178 | 171 | 7 | 0 |
| KIRREL | chr1:158064767 | c.G2131T | nonsyn. | p.A711S | 131 | 181 | 7 | 0 |
| ATF7 | chr12:53937094 | c.C284T | nonsyn. | p.A95V | 273 | 269 | 6 | 0 |
| ZNF726 | chr19:24116410 | c.G1492A | nonsyn. | p.A498T | 186 | 203 | 6 | 0 |
| PCDHB13 | chr5:140595593 | c.G1898A | nonsyn. | p.R633H | 292 | 242 | 6 | 0 |
| MCAT | chr22:43529240 | c.C982T | stopgain | p.Q328* | 269 | 246 | 5 | 0 |
| MUC17 | chr7:100678112 | c.A3415C | nonsyn. | p.S1139R | 368 | 355 | 5 | 0 |
| IGFN1 | chr1:201178696 | c.G4675C | nonsyn. | p.G1559R | 218 | 179 | 5 | 0 |
| TF | chr3:133467417 | c.A205G | nonsyn. | p.R69G | 228 | 196 | 5 | 0 |
| MUC17 | chr7:100678130 | c.G3433T | nonsyn. | p.A1145S | 399 | 364 | 4 | 0 |
| NOMO1 | chr16:14969038 | c.A2200C | nonsyn. | p.T734P | 439 | 429 | 2 | 0 |

**Appendix Table S2B. Insertions / deletions detected by exome sequencing of $VACO6_R$ cells compared to VACO6 cells.**

| GENE ID | Cohordinates | Insert / Del | Indel size | Effect | Position depth VACO6 | Position depth $VACO6_R$ | % altered reads | Occurrences in COSMIC |
|---|---|---|---|---|---|---|---|---|
| **AXIN1** | chr16:396233-396235 | Del | D-1 | frameshift | 248 | 127 | 26 | 1 |
| **AXIN1** | chr16:338207-338209 | Del | D-1 | frameshift | 438 | 357 | 25 | 1 |
| PCDH12 | chr5:141324955-141324956 | Insert | I-9 | in-frame | 101 | 60 | 57 | 17 |
| ATXN2L | chr16:28847349-28847351 | Del | D-1 | frameshift | 20 | 17 | 65 | 8 |
| BRD7 | chr16:50368677-50368680 | Del | D-2 | frameshift | 121 | 148 | 11 | 7 |
| EP400 | chr12:132547093-132547094 | Insert | I-9 | in-frame | 355 | 97 | 33 | 6 |
| EP400 | chr12:132547093-132547094 | Insert | I-6 | in-frame | 355 | 91 | 29 | 6 |
| OR2T2 | chr1:248616704-248616712 | Del | D-7 | frameshift | 428 | 370 | 5 | 5 |
| KCNN3 | chr1:154842199-154842200 | Insert | I-9 | in-frame | 221 | 28 | 61 | 4 |
| KCNN3 | chr1:154842199-154842200 | Insert | I-6 | in-frame | 221 | 24 | 54 | 4 |
| NOS1 | chr12:117703229-117703230 | Insert | I-1 | frameshift | 124 | 116 | 13 | 4 |
| OR8D4 | chr11:123777441-123777443 | Del | D-1 | frameshift | 182 | 117 | 9 | 4 |
| IFRD1 | chr7:112108038-112108040 | Del | D-1 | frameshift | 314 | 297 | 28 | 3 |
| NUMA1 | chr11:71746964-71746966 | Del | D-1 | frameshift | 139 | 119 | 8 | 3 |
| GCN1 | chr12:120595736-120595740 | Del | D-3 | in-frame | 231 | 198 | 5 | 3 |
| WRN | chr8:30945376-30945380 | Del | D-3 | in-frame | 304 | 283 | 4 | 3 |
| TMEM229A | chr7:123672456-123672460 | Del | D-3 | in-frame | 117 | 88 | 65 | 2 |
| KCNN3 | chr1:154842199-154842200 | Insert | I-9 | in-frame | 221 | 28 | 61 | 2 |
| KCNN3 | chr1:154842199-154842200 | Insert | I-6 | in-frame | 221 | 24 | 54 | 2 |
| HPS1 | chr10:100186986-100186987 | Insert | I-1 | frameshift | 159 | 164 | 30 | 2 |
| KCNN3 | chr1:154842199-154842200 | Insert | I-9 | in-frame | 221 | 28 | 61 | 1 |
| KCNN3 | chr1:154842199-154842200 | Insert | I-6 | in-frame | 221 | 24 | 54 | 1 |
| CLDN6 | chr16:3065485-3065487 | Del | D-1 | frameshift | 76 | 42 | 38 | 1 |
| MMP10 | chr11:102650394-102650395 | Insert | I-1 | frameshift | 208 | 183 | 34 | 1 |
| EP400 | chr12:132547093-132547094 | Insert | I-9 | in-frame | 355 | 97 | 33 | 1 |
| EP400 | chr12:132547093-132547094 | Insert | I-6 | in-frame | 355 | 91 | 29 | 1 |
| PRRC2A | chr6:31602640-31602642 | Del | D-1 | frameshift | 301 | 244 | 24 | 1 |
| ZNF717 | chr3:75786894-75786896 | Del | D-1 | frameshift | 879 | 118 | 24 | 1 |
| ZNF717 | chr3:75786555-75786556 | Insert | I-2 | frameshift | 737 | 107 | 19 | 1 |
| UNC93B1 | chr11:67771217-67771221 | Del | D-3 | in-frame | 231 | 152 | 7 | 1 |
| BHLHE22 | chr8:65494020-65494024 | Del | D-3 | in-frame | 221 | 179 | 6 | 1 |
| RPL14 | chr3:40503520-40503521 | Insert | I-3 | in-frame | 143 | 43 | 74 | -- |
| RPL14 | chr3:40503520-40503521 | Insert | I-6 | in-frame | 143 | 39 | 72 | -- |
| ATG3 | chr3:112253058-112253059 | Insert | I-1 | frameshift | 82 | 30 | 53 | -- |
| TRIL | chr7:28997596-28997597 | Insert | I-1 | frameshift | 143 | 99 | 46 | -- |
| RAPGEF6 | chr5:130764796-130764801 | Del | D-4 | frameshift | 157 | 149 | 46 | -- |
| EOMES | chr3:27763427-27763428 | Insert | I-6 | in-frame | 79 | 43 | 37 | -- |
| WI2-2373I1.2 | chr7:331127-331131 | Del | D-3 | in-frame | 33 | 41 | 37 | -- |
| ARV1 | chr1:231131566-231131569 | Del | D-2 | frameshift | 151 | 144 | 35 | -- |
| KIR2DL4 | chr19:55324674-55324675 | Insert | I-1 | frameshift | 253 | 157 | 35 | -- |
| BCAR1 | chr16:75269741-75269743 | Del | D-1 | frameshift | 65 | 54 | 33 | -- |
| MAML3 | chr4:140811063-140811079 | Del | D-15 | in-frame | 190 | 55 | 33 | -- |
| CWC22 | chr2:180810054-180810057 | Del | D-2 | frameshift | 122 | 105 | 30 | -- |
| SEC14L2 | chr22:30805433-30805435 | Del | D-1 | frameshift | 178 | 144 | 28 | -- |
| ARID1B | chr6:157099426-157099427 | Insert | I-6 | in-frame | 125 | 105 | 28 | -- |
| NRP2 | chr2:206562375-206562377 | Del | D-1 | frameshift | 263 | 211 | 27 | -- |
| GALNT5 | chr2:158167772-158167774 | Del | D-1 | frameshift | 80 | 87 | 24 | -- |
| CCDC159 | chr19:11461508-11461510 | Del | D-1 | frameshift | 103 | 105 | 20 | -- |
| ZNF880 | chr19:52887149-52887247 | Del | D-97 | frameshift | 110 | 54 | 19 | -- |
| HLA-A | chr6:29912028-29912031 | Del | D-2 | frameshift | 340 | 190 | 16 | -- |
| PMS1 | chr2:190670378-190670380 | Del | D-1 | frameshift | 219 | 182 | 12 | -- |
| ANKRD36C | chr2:96592972-96593062 | Del | D-89 | frameshift | 285 | 161 | 11 | -- |
| MUC5B | chr11:1267447-1271222 | Del | D-3774 | in-frame | 353 | 132 | 11 | -- |
| PRKRA | chr2:179301046-179307993 | Del | D-6946 | frameshift | 16 | 175 | 9 | -- |
| SLC10A5 | chr8:82606649-82606650 | Insert | I-1 | frameshift | 128 | 138 | 8 | -- |
| MAPKBP1 | chr15:42092079-42092081 | Del | D-1 | frameshift | 130 | 126 | 8 | -- |
| MUC5B | chr11:1267611-1271470 | Del | D-3858 | in-frame | 346 | 213 | 8 | -- |
| TLR4 | chr9:120476083-120476084 | Insert | I-1 | frameshift | 209 | 185 | 7 | -- |
| MUC5B | chr11:1267306-1271078 | Del | D-3771 | in-frame | 352 | 187 | 6 | -- |
| POTED | chr21:14982606-14982607 | Insert | I-111 | in-frame | 336 | 197 | 6 | -- |
| MUC12 | chr7:100637861-100642533 | Del | D-4671 | in-frame | 891 | 338 | 5 | -- |
| AXIN1 | chr16:354362-354364 | Del | D-1 | frameshift | 335 | 285 | 4 | -- |
| HRNR | chr1:152188231-152191052 | Del | D-2820 | in-frame | 1279 | 1261 | 2 | -- |
| MUC12 | chr7:100642276-100645526 | Del | D-3249 | in-frame | 1106 | 848 | 2 | -- |
| MUC12 | chr7:100643201-100646451 | Del | D-3249 | in-frame | 1092 | 818 | 2 | -- |
| MUC12 | chr7:100642062-100645312 | Del | D-3249 | in-frame | 1198 | 1364 | 2 | -- |
