# Supplementary material for: Loss of AXIN1 drives acquired resistance to WNT pathway blockade in colorectal cancer cells carrying RSPO3 fusions
Source: EMBO Mol Med. 2017 Jan 18;9(3):293–303. doi: 10.15252/emmm.201606773 (PMC5331210; doi:10.15252/emmm.201606773)
Supplement: Supplementary file 2 — Expanded View Figures PDF [file EMMM-9-293-s002.pdf]

Expanded View Figures

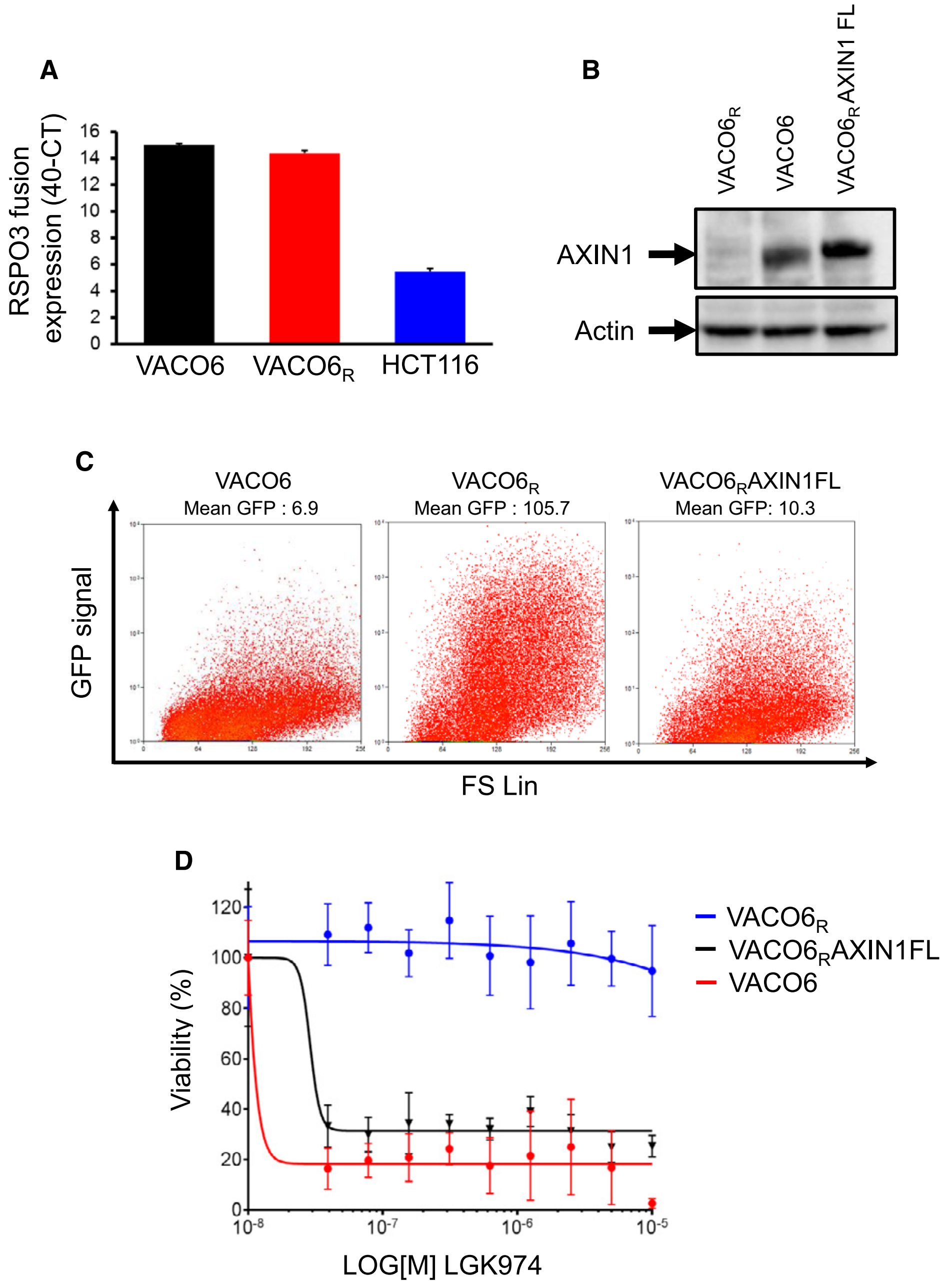

Figure EV1. Re-expression of the AXIN1 coding sequence re-sensitizes VACO6$_R$ cells to PORCN inhibition.

A qRT–PCR evaluation of PTPRK-RSPO3 fusion transcript expression in VACO6, VACO6$_R$, and HCT116 cells, as indicated. Data are expressed as means ± SD of three technical replicates from one representative experiment.

B Western blot showing AXIN1 protein expression in VACO6$_R$, VACO6, and VACO6$_R$AXIN1FL cells, as indicated.

C Flow cytometry analysis of the GFP signal in VACO6, VACO6$_R$, and VACO6$_R$AXIN1FL cells, all transduced with the 7xTcf-eGFP WNT reporter.

D ATP-based assay measuring viability, after 6 days of LGK974 treatment, of parental VACO6, VACO6$_R$, and VACO6RAXIN1FL cells, as indicated. Data are expressed as average ± SD of six technical replicates.

Source data are available online for this figure.

 © 2017 The Authors

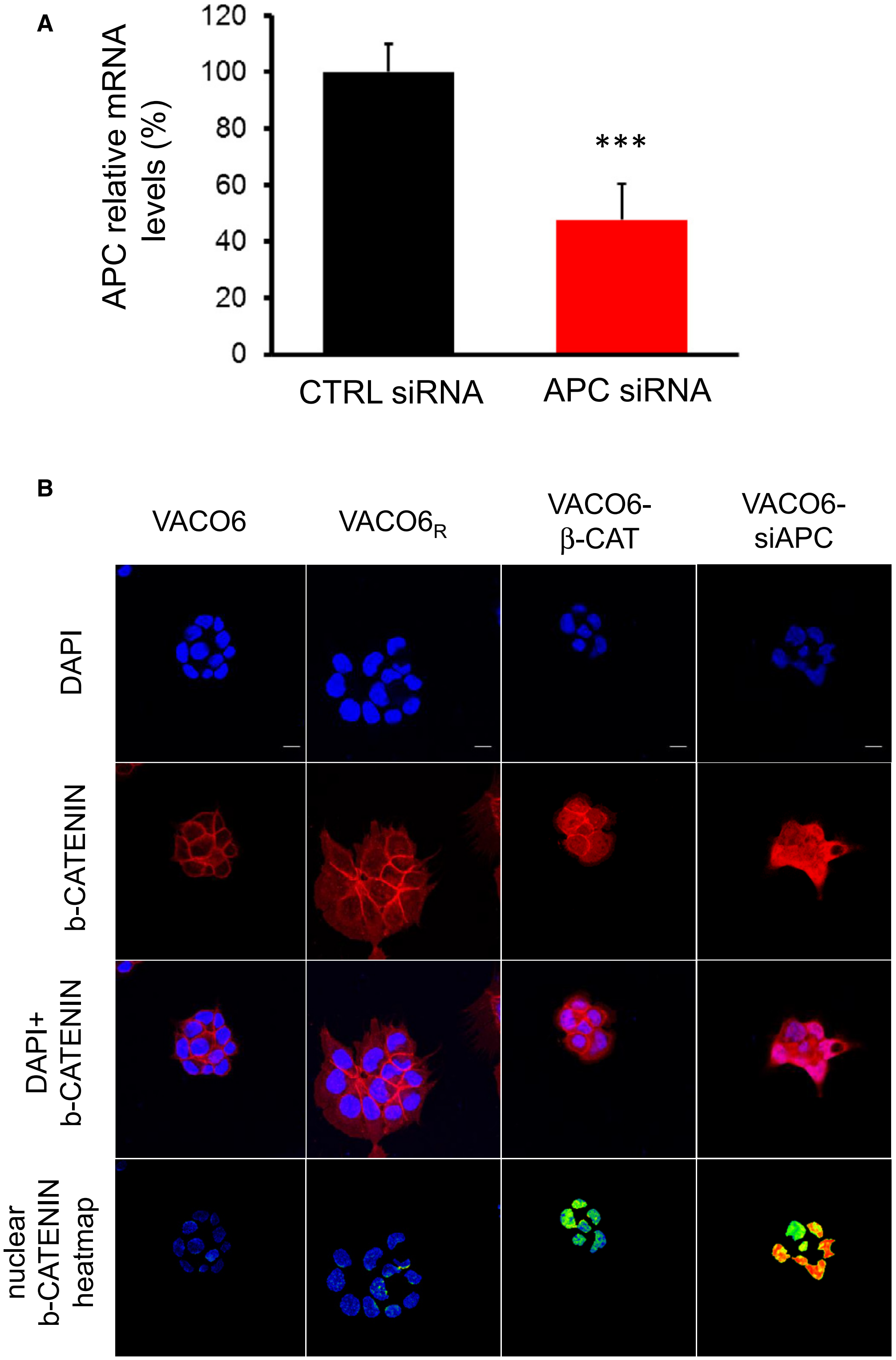

Figure EV2. Activated β-catenin transduction or transient APC downregulation in VACO6 cells induce increased nuclear β-catenin levels.

A qRT–PCR evaluation of APC mRNA expression in VACO6 cells transfected with control siRNA or APC siRNA, as indicated. Two-sided Student's *t*-test, ****P*-value < 0.001, $P = 8.24E{-}04$. Data are expressed as means ± RMSE of three technical replicates from one representative experiment.

B Representative confocal microscopy images of VACO6 cells (as indicated) showing β-catenin distribution and GFP expression. Nuclei are stained with DAPI (blue). Scale bars, 10 μm.

© 2017 The Authors

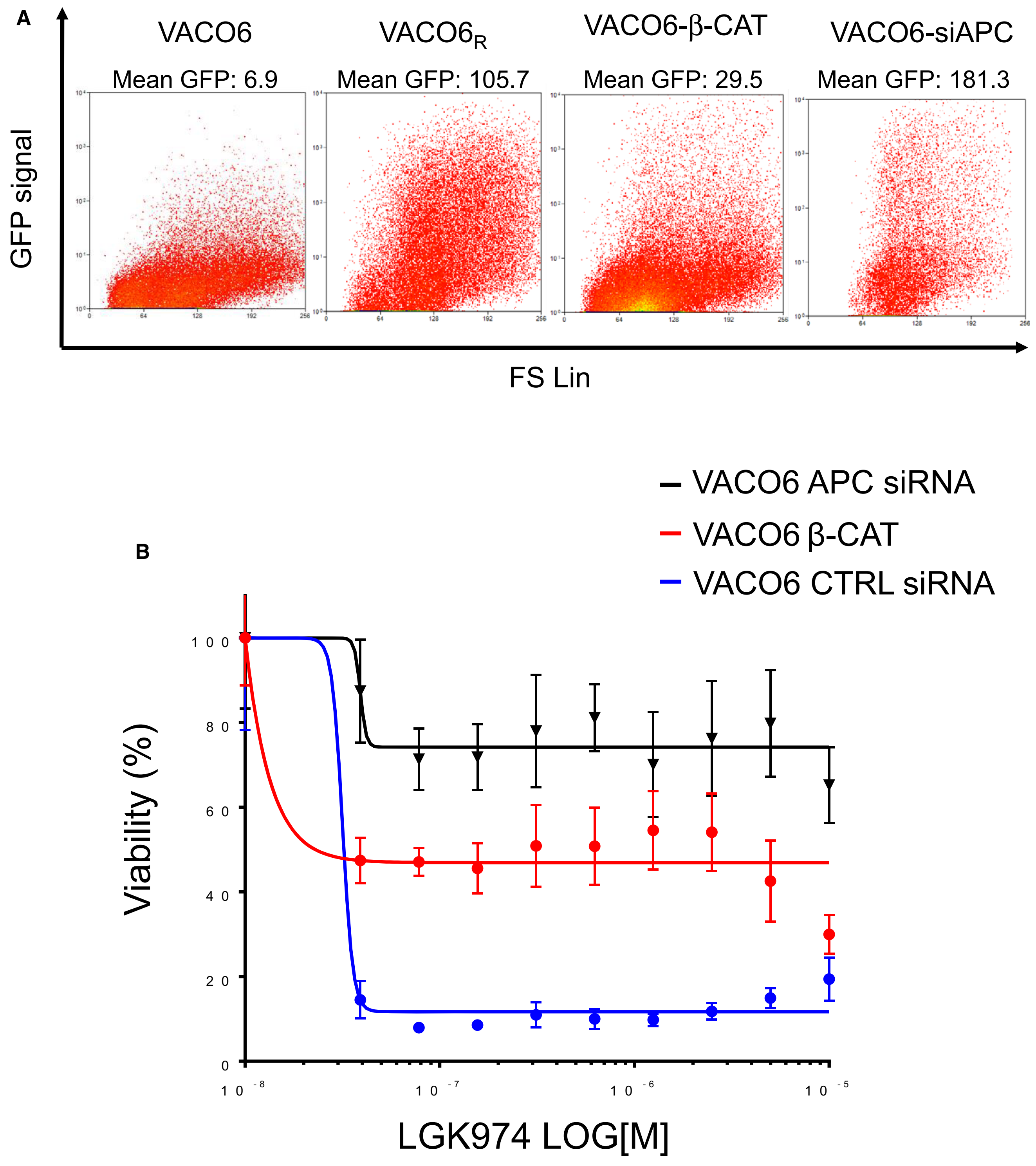

Figure EV3. Activated β-catenin transduction or transient APC downregulation increase WNT pathway activity and induce resistance to WNT pathway inhibitors in VACO6 cells.

A Flow cytometry analysis of GFP fluorescence in VACO6, $VACO6_R$, VACO6-b-CAT, and VACO6-siAPC, as indicated, all transduced with the 7xTcf-eGFP WNT reporter.

B ATP-based assay measuring viability, after 7 days of LGK974 treatment, of VACO6 cells transfected with control siRNA or with APC siRNA, or transduced with activated β-catenin. Data are expressed as average ± SD of six technical replicates from one representative experiment.

 © 2017 The Authors
