# Supplementary figures and images for: Loss of AXIN1 drives acquired resistance to WNT pathway blockade in colorectal cancer cells carrying RSPO3 fusions
Source: EMBO Mol Med. 2017 Jan 18;9(3):293–303. doi: 10.15252/emmm.201606773 (PMC5331210; doi:10.15252/emmm.201606773)

Appendix Figure S6A

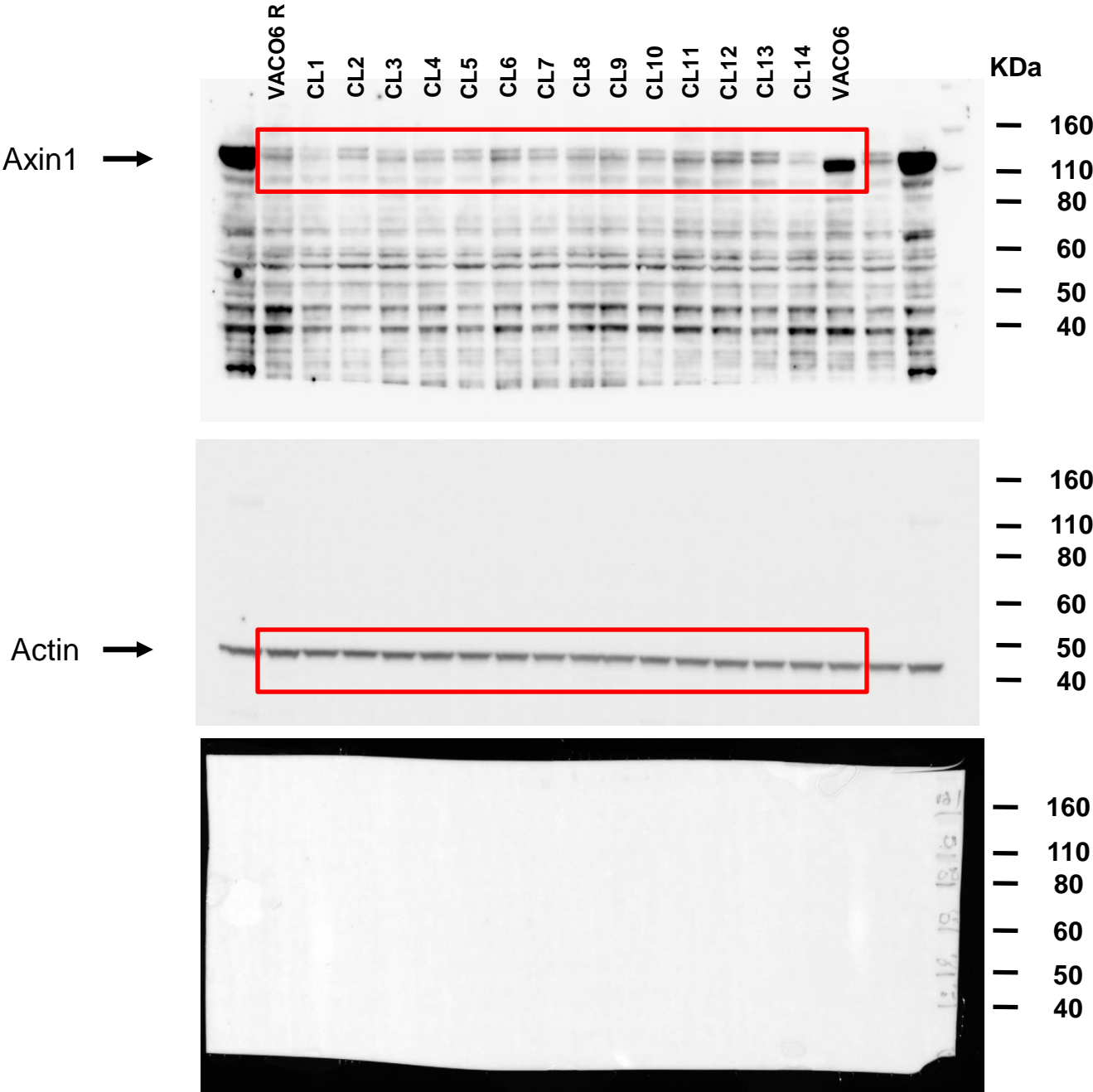

Supplement: Supplementary file 3 — Source Data for Expanded View and Appendix [file EMMM-9-293-s004.zip › Source_Data_for_ExpandedView_and_Appendix/Source_Data_Appendix_Figure_S6A.pdf]

Figure EV1 B

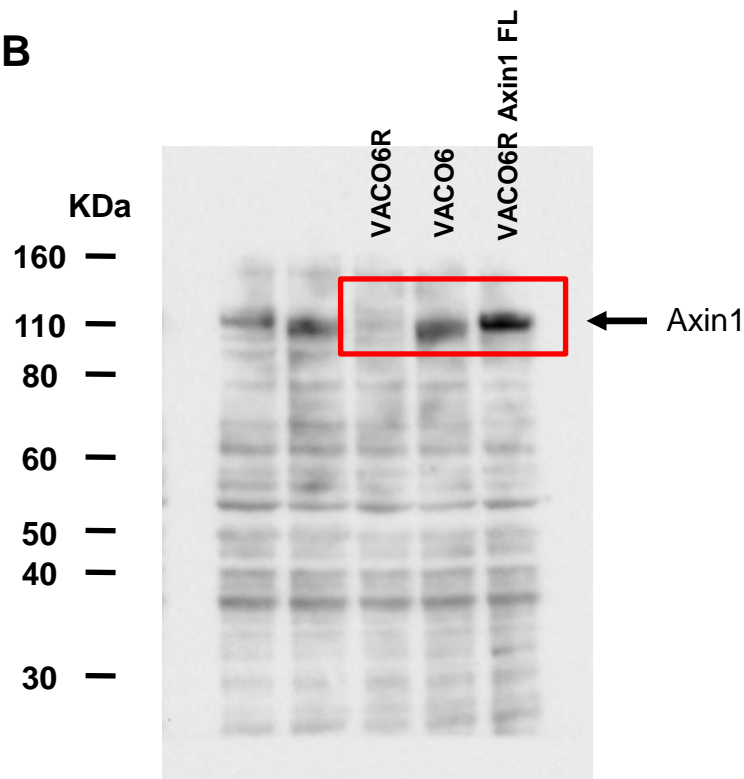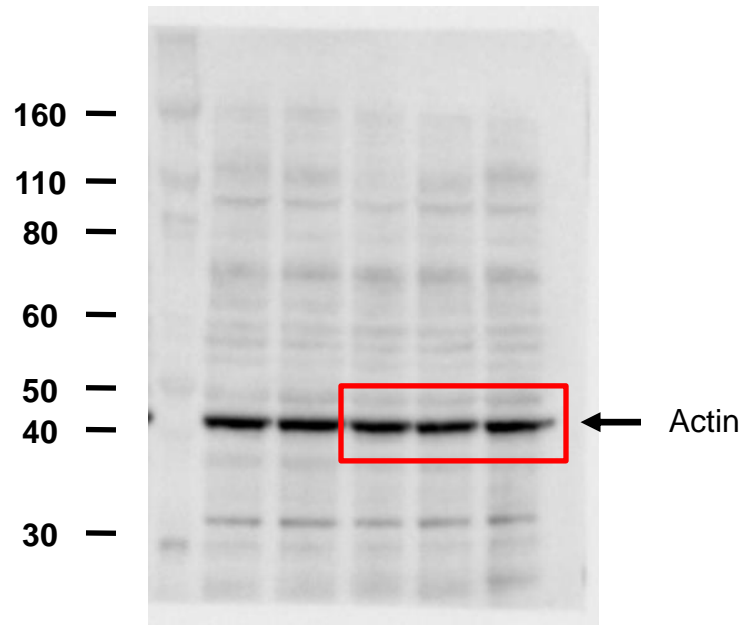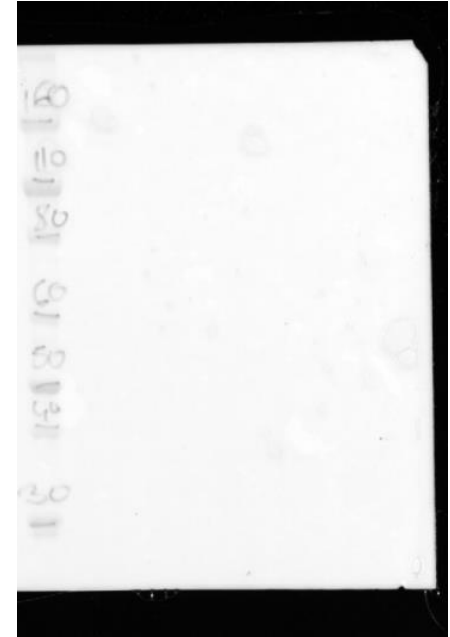

Supplement: Supplementary file 3 — Source Data for Expanded View and Appendix [file EMMM-9-293-s004.zip › Source_Data_for_ExpandedView_and_Appendix/Source_Data_Figure_EV1B.pdf]
