# Supplementary material for: Loss of AXIN1 drives acquired resistance to WNT pathway blockade in colorectal cancer cells carrying RSPO3 fusions
Source: EMBO Mol Med. 2017 Jan 18;9(3):293–303. doi: 10.15252/emmm.201606773 (PMC5331210; doi:10.15252/emmm.201606773)
Supplement: Supplementary file 5 — Source Data for Figure 3 [file EMMM-9-293-s003.pdf]

Figure 3

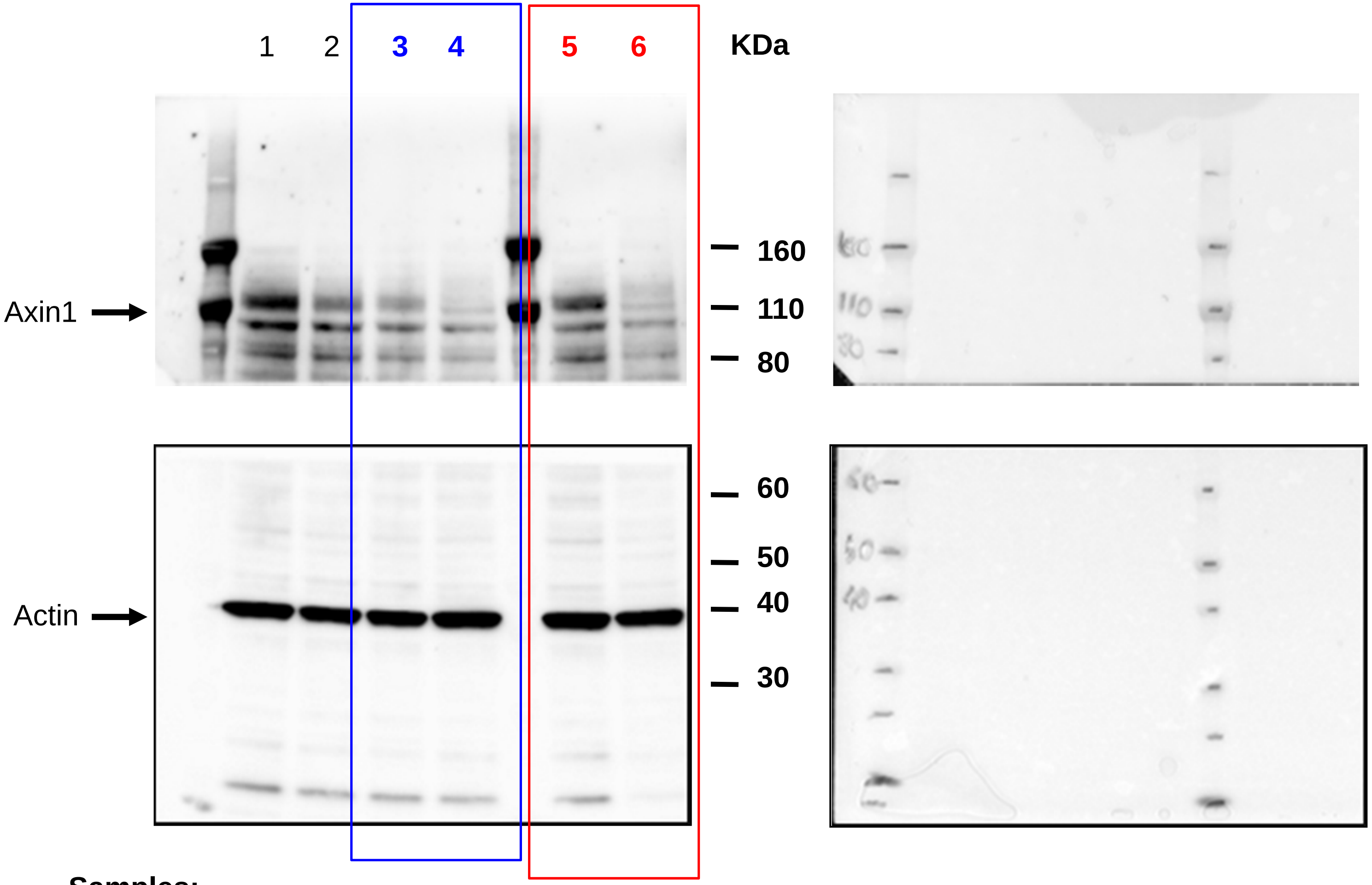

Samples:

1 VACO6 WT
2 VACO6 Mock
3 VACO6 CTRL- (non-silencing)
4 VACO6 siRNA Axin1
→ Figure 3E
5 VACO6 WT
6 VACO6$_R$
→ Figure 3C
